# Disease Burden, Risk Factors, and Temporal Trends in Breast Cancer in Low‐ and Middle‐Income Countries: A Global Study

**DOI:** 10.1002/puh2.223

**Published:** 2024-07-29

**Authors:** Mingjun Gao, Sofia Laila Wik, QinYao Yu, FanYu Xue, Sze Chai Chan, Shui Hang Chow, Yusuff Adebayo Adebisi, Claire Chenwen Zhong, Don Eliseo Lucero‐Prisno, Martin CS Wong, Junjie Huang, Global Health Focus Epidemiology Group (GHFEG)

**Affiliations:** ^1^ The Jockey Club School of Public Health and Primary Care Faculty of Medicine The Chinese University of Hong Kong Hong Kong SAR China; ^2^ Adam Smith Business School College of Social Science University of Glasgow Glasgow UK; ^3^ Karolinska Institute Stockholm Sweden; ^4^ Jinan University–University of Birmingham Joint Institute, Jinan University Guangzhou China; ^5^ School of Mathematics College of Engineering and Physical Sciences University of Birmingham Birmingham UK; ^6^ Faculty of Health Sciences University of Ottawa Ottawa Canada; ^7^ Nuffield Department of Population Health University of Oxford Oxford UK; ^8^ Department of Global Health and Development London School of Hygiene and Tropical Medicine London UK; ^9^ Centre for Health Education and Health Promotion Faculty of Medicine The Chinese University of Hong Kong Hong Kong SAR China

**Keywords:** breast cancer, disease burden, low‐ and middle‐income countries, risk factors, temporal trends

## Abstract

**Introduction:**

Breast cancer poses significant health risks to women and strains healthcare systems extensively. In low‐ and middle‐income countries (LMICs), limited resources and inadequate healthcare infrastructures further exacerbate the challenges of breast cancer prevention, treatment, and awareness.

**Methods:**

We examined the prevalence, risk factors, and trends of breast cancer in LMICs. Data on disability‐adjusted life years (DALYs) and breast cancer risk factors were extracted from the Global Burden of Disease (GBD) databases for 203 countries or territories from 1990 to 2019. LMIC DALY rates were examined using joinpoint regression analysis.

**Results:**

Among the income groups, the lower middle‐income category had the highest DALYs value, with 1787 years per 100,000 people. LMICs collectively accounted for 74% of the global burden of DALYs lost due to breast cancer in 2019. However, it remained relatively consistent in lower middle income countries (LMCs). In LMCs, the risk associated with metabolic syndromes was higher compared to that with behavioral factors alone. For the past three decades, breast cancer incidences increased significantly in LMCs (average annual percent change [AAPC]: 1.212, confidence intervals [CI]: 1.51–1.87, *p* < 0.001), upper middle income countries (AAPC: 1.701, CI: 1.12–1.48, *p* < 0.001), and low‐income countries (AAPC: 1.002, CI: 1.57–1.68, *p* < 0.001).

**Conclusion:**

This research shows how breast cancer in LMICs is aggravated by low resources and healthcare infrastructure. To effectively combat breast cancer in these areas, future strategies must prioritize improvements in healthcare infrastructure, awareness campaigns, and early detection mechanisms.

## Graphical abstract

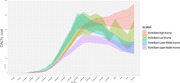

## 1 Introduction

Research on disease burden shows that breast cancer presents a significant challenge for countries at all income levels around the world. Moreover, cancer is the most frequent disease in low‐ and middle‐income countries (LMICs), making the burden of the disease particularly pronounced [1, 2]. Breast cancer has proved to be one of the most common cancer diseases, which has a significant menace for females [3]. This disease exerts its impact on all segments of a society [4]. From social and individual perspectives, breast cancer engenders opportunity costs, productivity losses, and compromises to human health [5]. In decision level perspective, some previous studies have suggested that health resource constraints in LMICs contribute to the increased disease burden of breast cancer [6, 7]. The pressure of allocating resources at the decision‐making level is reflected in this. The availability of medical resources in LMICs is inherently constrained by the relatively modest economic conditions prevailing in these nations [2]. As such, it becomes imperative to accurately identify the disease burden, risk factors, and temporal trends associated with breast cancer to facilitate effective treatment, prevention, and the equitable allocation of healthcare resources [8].

On the basis of the importance of breast cancer research, this article aims to conduct a comprehensive study that will provide valuable information to help policymakers develop appropriate strategies. Specifically, it will discuss how and why the dynamics of the breast cancer burden are changing in LMICs, and how understanding these dynamics can lead to effective mitigation and management strategies. To this end, three key elements will be employed in this study. First, longitudinal data analysis will be conducted on various age groups and geographic locations to assess the disease burden in terms of disability‐adjusted life years (DALYs). This endeavor will provide a comprehensive overview of the global scenario. Second, temporal trends in breast cancer incidence and mortality rates spanning the period from 1990 to 2019 will be scrutinized to ascertain whether recent influential factors, such as policies or environmental changes, have exerted a positive or negative influence on these rates. Finally, the analysis of risk factors will shed light on the specific threats posed by breast cancer within different income groups.

Ultimately, the aim of this study is to gain a deep and comprehensive understanding of breast cancer in LMICs, thereby facilitating the implementation of targeted measures to reduce unnecessary losses and optimize resource allocation in these constrained settings.

## 2 Materials and Methods

### 2.1 Database and Materials

The Global Burden of Disease (GBD) [9] serves as the primary database in this study. GBD is the database that comprehensively documents the costs of diverse diseases, employing varied measurements spanning multiple countries and temporal periods. The extraction of DALYs cost, risk factors, and incidence/death rates related to breast cancer for each income group resulted in the inclusion of 203 countries or territories in the GBD data spanning from 1990 to 2019. Because male breast cancer is relatively rare, its occurrence is not statistically significant for the purposes of this study. Therefore, it is not addressed in this article.

The World Bank's (WB's) [10] income group categorization is employed as a reliable benchmark for classification purposes. The WB offers an extensive array of economic information, characterized by its relative accuracy. Consequently, this research will adopt the income classification provided by the WB.

This study encompasses a range of demographic information, including population structures. To meet the research requirements, data from reputable sources, such as the Pew Research Center (PRC) [11] and the 2022 Revision of the World Population Prospects by the United Nations (UN) [12], were referenced. Accordingly, this research utilized selected demographic information sourced from these databases.

### 2.2 Disease Burden

To gain a comprehensive understanding of the global burden of breast cancer, DALYs are a comparable unit of measure that combines years lost due to premature death and years lived with disability [13]. DALYs serve as one of the main references at the decision‐making level (e.g., World Health Organization [WHO]), and people's health levels can also be found from DALYs [1]. Consequently, this study will utilize DALYs for each age group and country, as derived from the GBD database [9], to differentiate among income levels as classified by the WB [10], namely upper‐middle‐income countries (UMCs), lower‐middle‐income countries (LMCs), and low‐income countries (LCs).

Age group analysis was conducted using line graphs with 95% confidence intervals (CI). These graphs can assist this study and policymakers in comprehending the distribution of health problems and disease burdens faced by individuals in different age groups. Specifically, data from DALYs of all ages in 2019 will be used, where the age bands for this study are set to be every 5 years. In order to point out the problems faced by different types of countries, the study will use four bands on the basis of income levels.

Geographic analysis involves referring to the DALYs crude rate and age‐standardized rate (ASR) for 203 countries or regions on a map, while considering income groups and demographics information via PRC [11] and UN [12] as well. In this research, the aim is to compare the DALYs calculated using the crude rate and the ASR to understand the distribution of patient age ranges. Countries will be color‐coded on the basis of the comparison between the crude rate and ASR. If the crude rate is higher than the ASR, countries will be represented in blue, whereas if the crude rate is lower than the ASR, countries will be presented in orange. This approach allows backward extrapolation on the basis of the ASR's arithmetic rules to derive the width of the age distribution of DALYs in each country. The income groups (UMCs, LMCs, and LCs) will be visualized on a map, with each country's color representing its income group, ranging from light to dark. The study aims to identify the correlation between DALYs and demographic and geographic characteristics by categorizing different types of cases. Maps will be utilized to explore these relationships.

### 2.3 Temporal Trends Analysis

Temporal trends will examine the correlation between external factors (e.g., policies, environment, or income) and DALYs. This analysis will involve studying the incidence and death rates over the past three decades, using data extracted from the GBD database [9]. Cause of that incidence rates can be used to understand the prevalence and trends of cancer in a population, and death rates can reflect the extent to which cancer threatens the lives of the population. For this reason, the extracted data will be analyzed using Joinpoint Regression Software to determine the annual average percent change (AAPC) and average percent change (APC) and identify any inflection points in the trends [14]. The Joinpoint Regression analysis program was developed as a component of the National Cancer Institute of the United States’ Surveillance, Epidemiology, and End Results (SEER) Program. Additionally, tables and graphs of trends will be explored for each income group to understand the external influences.

### 2.4 Risk Factor Trend Analysis

Risk factor analysis will be undertaken by leveraging data sourced from the GBD database existing on LMICs [9]. According to GBD's taxonomy of risk factors [15], they are categorized into three orders: main category, subcategory, and subgroup according to top to bottom categorization affiliation. It is anticipated that each risk factor (eTable 1) will exhibit a distinct value within respective income groups. Such proportions will yield valuable insights into the heterogeneous risk levels present across diverse income groups. In order to determine the development of long‐term trends, this study will use risk factor data for the period from 1990 to 2019 to form a trend map.

### 2.5 Ethical Considerations

This study utilized secondary data publicly available from GBD, specifically existing datasets related to the burden of breast cancer. As such, these data sources have been subjected to appropriate ethical approval and consent processes at the time of initial data collection. Detailed information about the original data collection protocols, including ethical approvals, are noted in the data source organizations.

In addition, this research team ensured that ethical standards for secondary data analysis were adhered to and intuitional review board for ethical approval was obtained from the Chinese University of Hong Kong (No. SBRE‐22‐0826). This includes adhering to the principles of fair use and ensuring that secondary analyses add value to existing knowledge without compromising ethical or scientific integrity.

## 3 Results

### 3.1 Disease Burden

#### 3.1.1 Age Group Analysis

**FIGURE 1:**
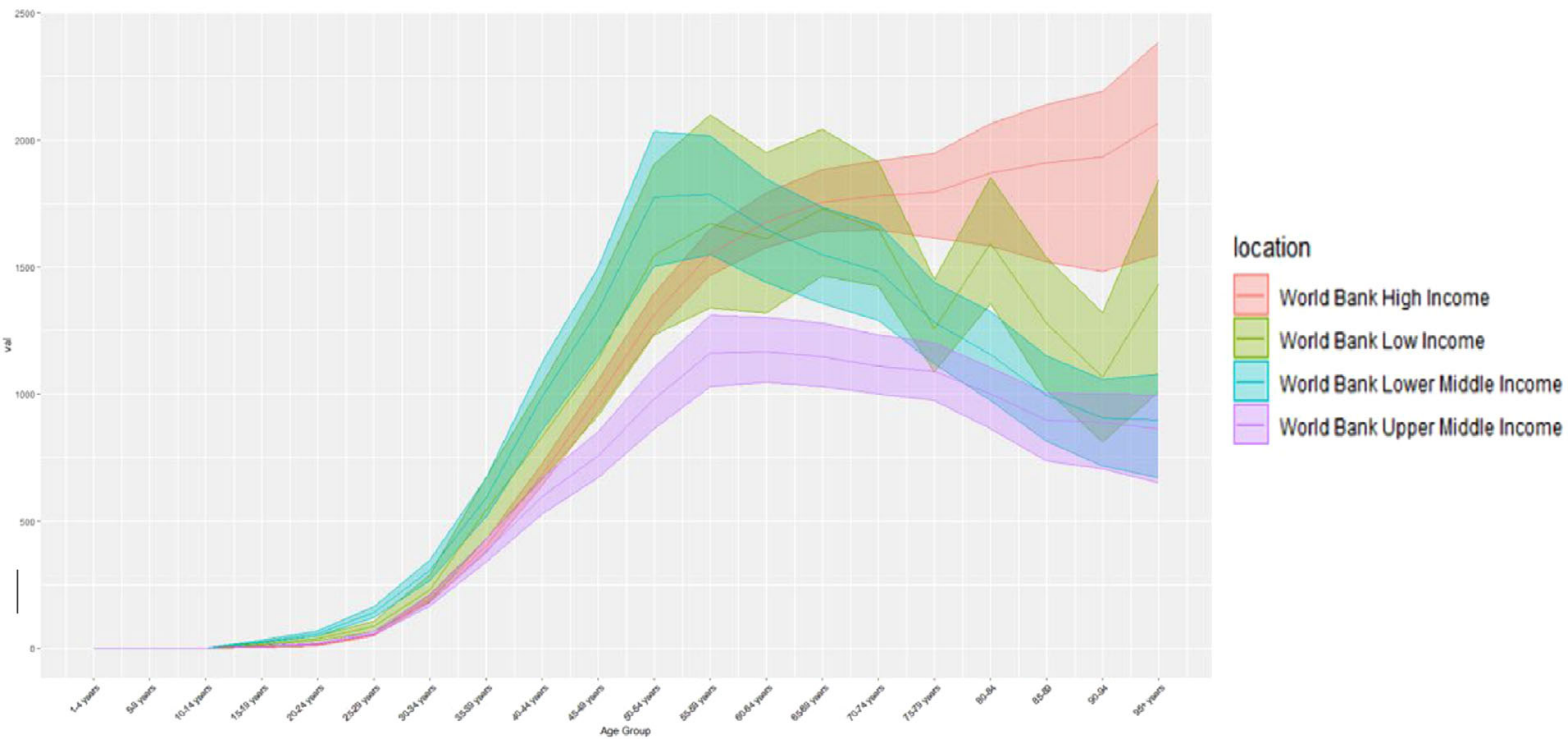
DALYs cost in each age group, one group for five. Twenty groups total. DALYs, disability‐adjusted life years.

In the present study (Figure 1), our findings reveal that LMCs exhibit the most substantial burden of disease within themselves, as indicated by the relatively greatest cost for DALYs in most age groups (14/20). Between age 65 and 80–84 years, LMCs have maintained the highest cost of DALYs in each age group. LC's DALYs cost was consistently higher than UMC's, becoming the highest cohort cost at ages 65–74, 80–95+ years.

The DALYs peak of LMC burden occurs in the population aged 50–54 years, which is 1776.45 per 100,000 individuals annually. Subsequently, the population aged 55–59 years in UMCs experience a peak immediately following the LMC peak which is 1310.93 DALYs cost per 100,000 individuals per year. The peak of LC is observed in the population aged 65–69 years, which is 1728.32 per 100,000 individuals per year.

#### 3.1.2 Geographic Analysis

**FIGURE 2:**
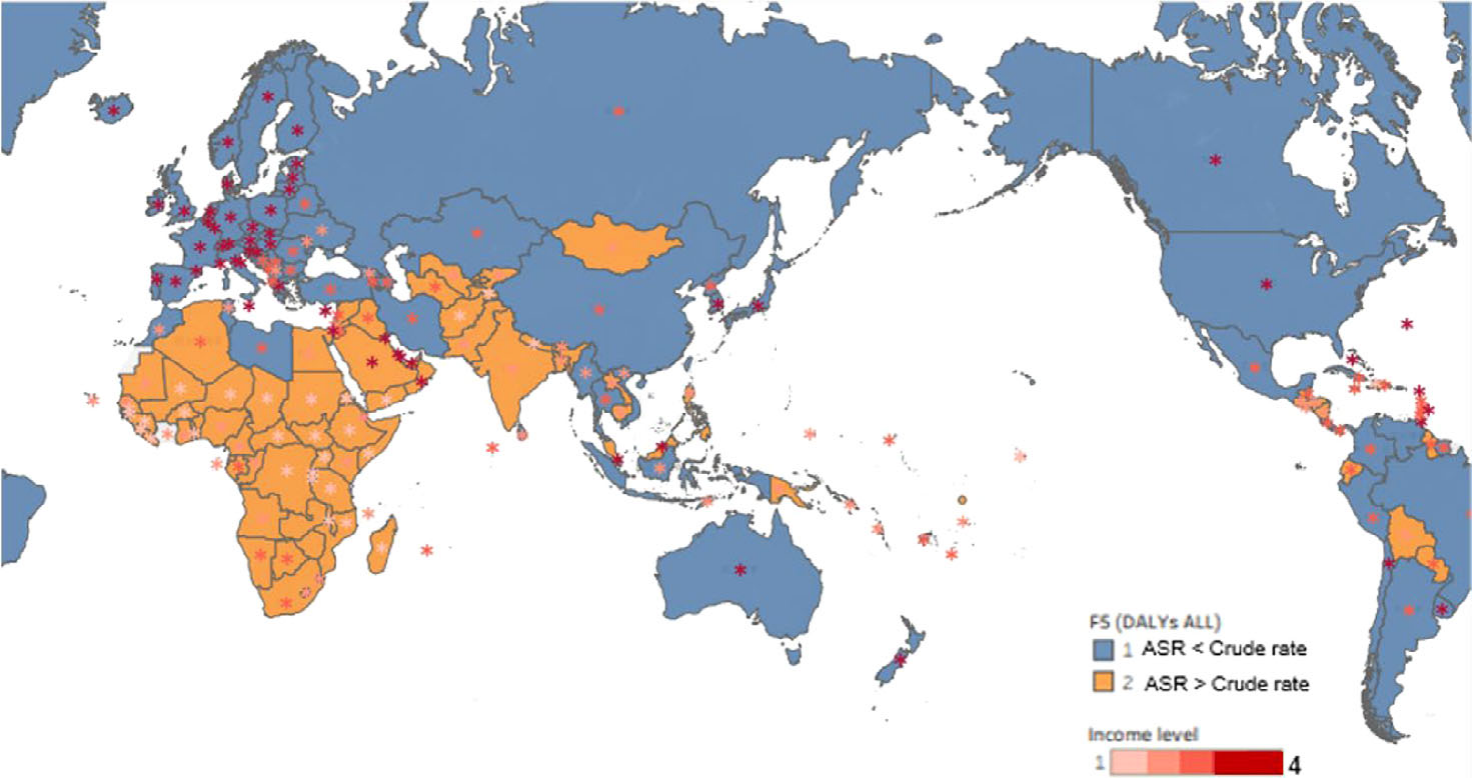
Competition between age‐standardized rate and crude rate with income information in worldwide.

There was geographic research (Figure 2), and out of a total of 203 areas (*n* = 203), 99 areas (49%) are indicated by a color on the map to represent ASR greater than the crude rate in a country, representing as the orange. Conversely, 104 areas (51%) are represented in blue, indicating that the ASR was lower than the crude rate. Derived backward from ASR's formula, blue countries imply a relatively stable distribution of DALYs cost across an age class. Orange means that DALYs cost is distributed over a wider age span.

The distribution of “orange” areas varies considerably from a continental perspective (eTable 2), and 87% of African countries faced situations with ASR > crude rate. This percentage was 81% for Oceania, 56% for Asia, 27% for America, and 0% for Europe. In addition, 90.9% of “orange” countries are LMICs that are shown in Figure 2, with a 100% “orange” coverage rate in LCs, 76% in LMCs, 34% in UMCs, and 16% in high‐income countries (HCs). Additionally, the “orange” regions among the HCs include Bahrain, Kuwait, Kyrgyzstan, Malaysia, Oman, Palestine, Qatar, Saudi Arabia, and the United Arab Emirates, all of which are Middle Eastern countries.

### 3.2 Temporal Trends

#### 3.2.1 Death

**FIGURE 3:**
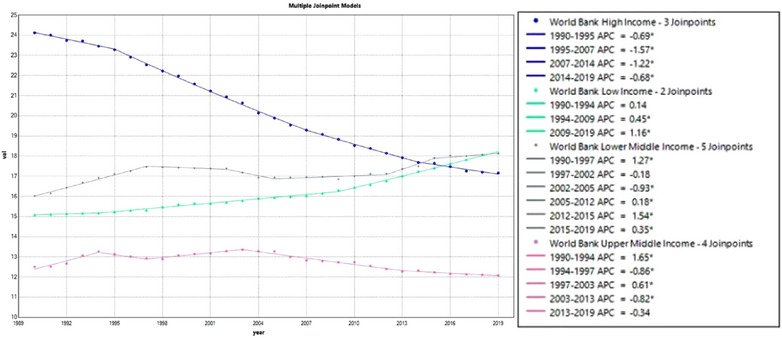
AAPC of breast cancer death rate trends, 1990–2019. AAPC, average annual percent change.

Death rate trends are shown by Joinpoint Regression (Figure 3; eTable 3). This section focuses on death trends. UMCs show five segments. There were positive and negative fluctuations for three times, which occurred among segments 1–4. The biggest positive APC fluctuation is 1.646 (*T* = 1.294; CI: 1.07–2.22; *p* = 0.209), and the biggest negative APC fluctuation is −0.863 (*T* = 27.123; CI: −2.62 to 0.93; *p* < 0.001). The AAPC of UMCs is −0.092 (*T* = −0.836; CI: −0.31 to 0.12; *p* = 0.403). LMs demonstrate a general trend of upward movement. There are six segments, which is the most frequent number of inflection points among income groups. The segments from 1997 to 2002 and 2002 to 2005 show only downward trends. The AAPC of LMs is 0.429 (*T* = 5.333; CI: 0.27–0.59; *p* < 0.001). LCs demonstrate an upward trend in three segments. Segment 3 has the biggest APC, which is 1.164 (*T* = −2.403; CI: 1.10–1.22; *p* = 0.029). AAPC is 0.654 (*T* = 32.502; CI: 0.61–0.69; *p* < 0.001).

#### 3.2.2 Incidence

**FIGURE 4:**
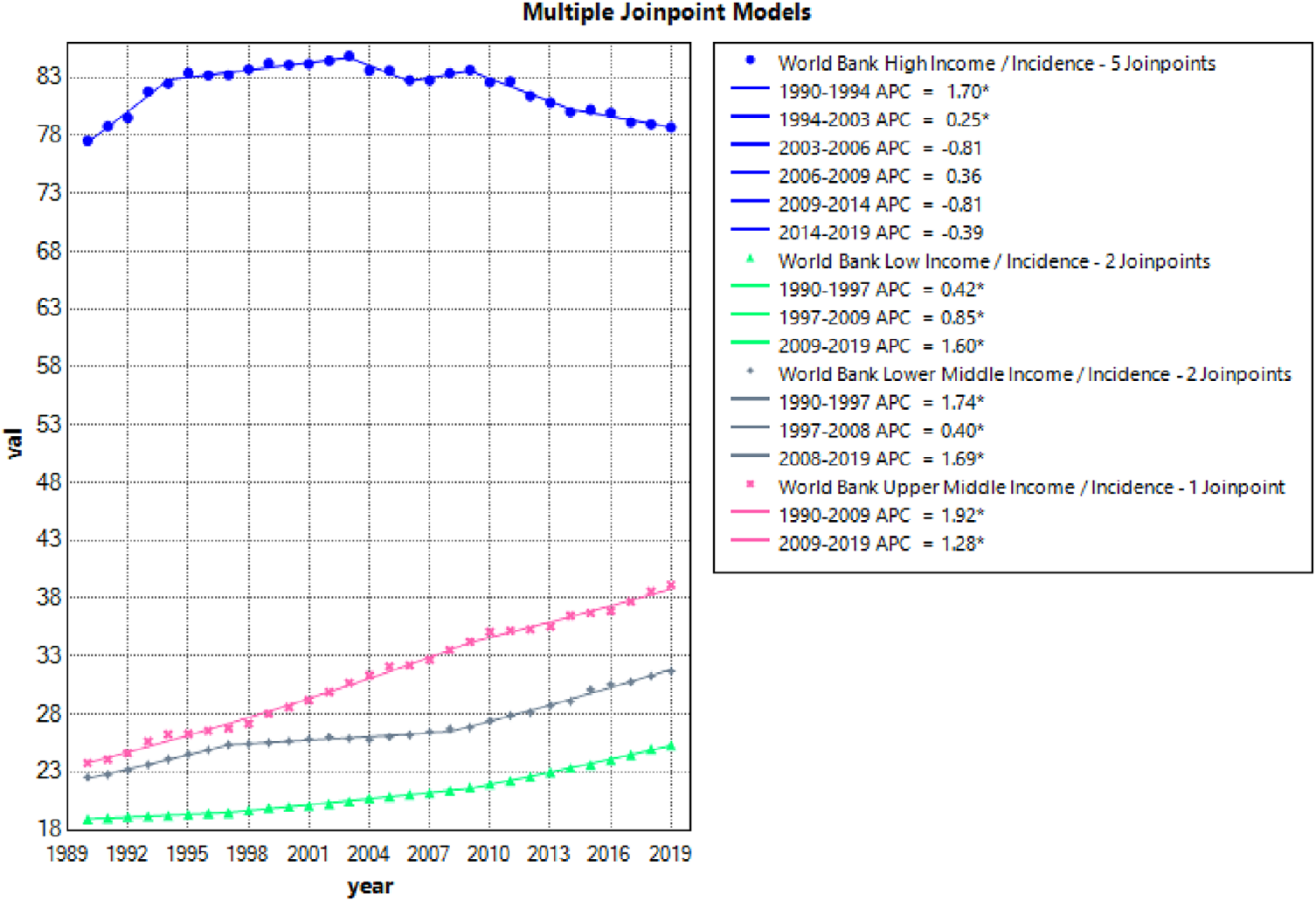
AAPC of breast cancer incidence rate trends, 1990–2019. AAPC, average annual percent change.

The incidence rate was also observed using Joinpoint analysis (eTables 4 and 5; Figure 4). In this study, UMCs displayed the highest fluctuation in AAPC, reaching 1.701 (*T* = 34.696; CI: 1.12–1.48; *p* < 0.001). Moreover, each segment of UMCs maintained upward APCs. Similarly, LMCs and LCs experienced an upward trend in the incidence rate. LMCs’ AAPC was 1.212 (*T* = 33.915; CI: 1.51–1.87; *p* < 0.001), whereas LCs’ AAPC was 1.002 (*T* = 58.178; CI: 1.57–1.68; *p* < 0.001). Both LCs and LMCs exhibited three segments of separation, with the timing of separation being nearly synchronous, adding to the coincidental nature of their findings.

### 3.3 Risk Factors

#### 3.3.1 Main Category

**FIGURE 5:**
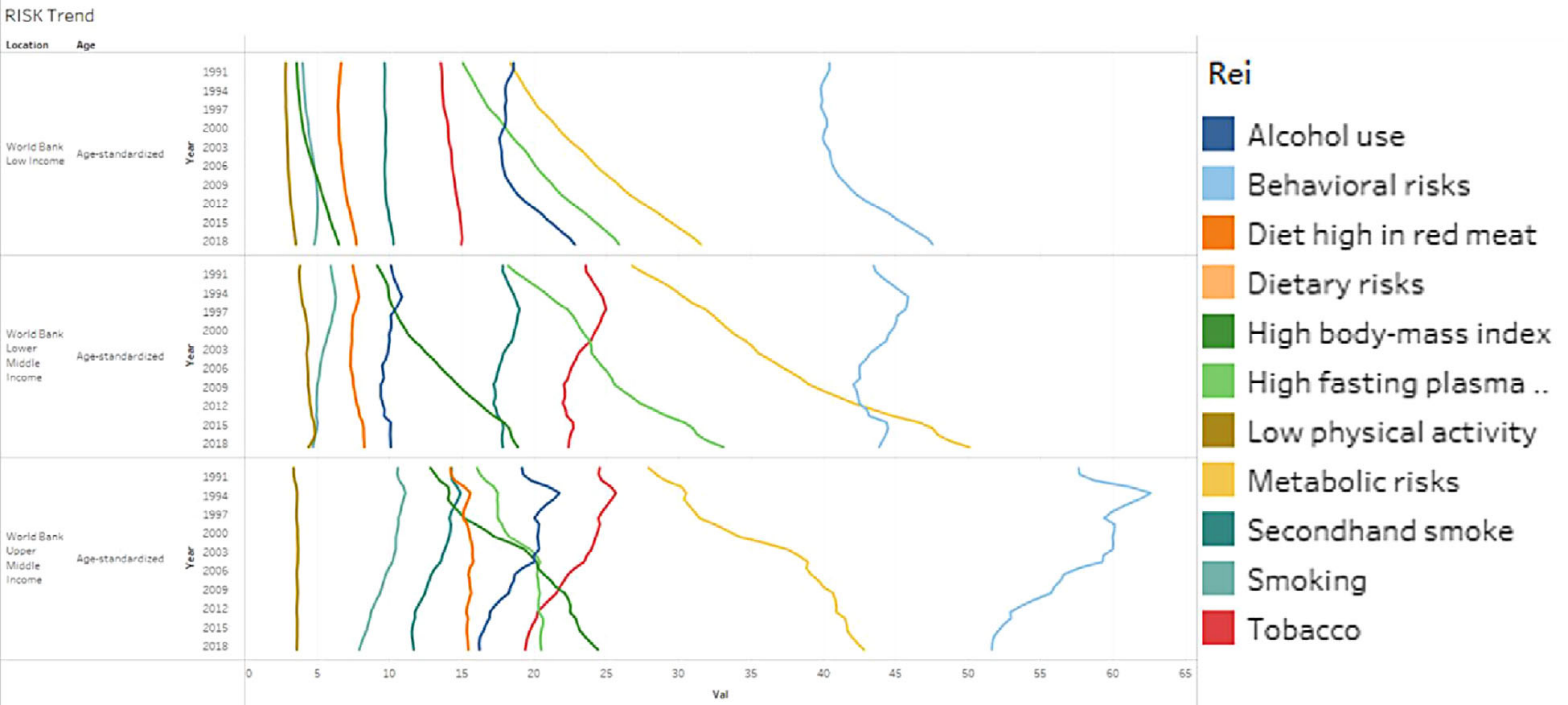
Risk factor trends, 1990–2019.

The main category analysis encompasses two key risk factors: behavioral and metabolic, as presented in eTable 1. Moreover, the trends of risk factors are shown by Figure 5. Over the past three decades, both UMCs and LCs have exhibited a higher proportion of behavioral risk. Similarly, in LMCs, behavioral risk dominated the risk proportion prior to 2013. However, in the 2013 data, metabolic risk replaced behavioral risk as the dominant factor in terms of proportional risk.

Regarding the trends observed, UMCs’ behavioral risk demonstrates a declining trend with a 10.4% decrease from 1990 to 2019. Conversely, LCs exhibit a rising trend in behavioral risk with 17.7% total change rate. LMCs, on the other hand, largely maintain the original pattern, the total change rate of which is <1%. In terms of metabolic risk, all three groups demonstrate an upward trend, indicating a consistent increase in the prevalence of metabolic risk factors.

#### 3.3.2 Subcategory

The trend of low physical activity is observed consistently across the three income groups. Similarly, the dietary risk trends mirror those of low physical activity. However, it should be noted that the APC for low physical activity is expected to be higher in UMCs compared to other countries.

Moreover, the prevalence of high fasting plasma glucose (HFPG) and high body mass index (BMI) demonstrates an upward trend in three income groups. In the case of HFPG, LMCs have experienced a significant increase with 85.42% total change rate. On the other hand, UMCs experienced a notable reduction in growth, with the rate approaching 0 by 2005. The change rate from 2005 to 2019 is 1.8%. As for high BMI, LCs exhibit relatively slower growth rates.

Regarding alcohol use and tobacco, there is a high degree of correlation observed in both LMCs and UMCs. However, alcohol use shows relatively more pronounced fluctuations in LCs.

#### 3.3.3 Subgroup

Smoking and passive smoking have the same trend in LMCs and UMCs. However, there is a negative correlation in LCs. For individuals consuming high amounts of red meat, levels of LCs and LMCs exhibit a pattern of decline followed by an increase, occurring approximately at the same time. However, UMCs have significant fluctuations as well as maintain an increasing trend.

## 4 Discussion

### 4.1 Key Findings

#### 4.1.1 Geocultural Factors

Geocultural factors may potentially have an indirect association with the incidence of breast cancer. The current study (Figure 2) reveals a contrasting relationship between the ASR and the crude rate of DALYs of the breast cancer. The ASR calculated using an average weight formula [16] indicates that if it exceeds the crude rate, it signifies that the loss of DALYs is widely distributed in all age groups [17]. In 91.67% of countries in the Middle East and Africa, enhancing quality‐adjusted life years (QALYs) is challenging due to the natural aging process of the human body [18]. For these reasons, when ASR surpasses the crude rate, it suggests that certain countries may be unable to provide effective medical assistance, even for relatively young and easily treatable patients. Else, it may mean that certain countries have a risky population structure.

This article proposes two potential explanations for this scenario: First, the patient may be diagnosed with breast cancer that is already in intermediate to advanced stages. The country may have insufficient medical resources to offer standardized treatment. Moreover, few research studies prove that some gender bias may exist in “poor” counties [19]. Second, the demographic composition of these countries is mostly young, potentially leading to a crude rate smaller than the ASR [12]. Under this premise, the article finds that the HC group in Middle Eastern countries also encounters a situation where the ASR exceeds the crude rate. Combining with the significant geographical overlap among the Middle East, Africa, and the “orange” scenario, the article suspects that resource distribution is unequal in LMICs. Gender, income, and faith may serve as underlying factors contributing to this inequality. Consequently, this article suggests that the phenomenon of ASR exceeding the crude rate may implicitly be linked to geocultural factors.

#### 4.1.2 Risk Factors

These findings suggest that behavioral risk factors have been a dominant contributor to overall risk, but metabolic risk factors are gaining prominence in recent years in LMCs. HFPG plays a crucial role in the rising of metabolic risk, with a more pronounced impact observed LMCs. This study proposes a hypothesis suggesting that LMCs might encounter heightened subhealth [20], as HFPG can potentially arise from diabetic complications. In addition, there is also a correlation between the rise in HFPG and genetic and environmental conditions [21]. Notably, evident inflection points in HFPG prevalence occurred in UMCs in 2013. If this aligns with the aforementioned speculation, it could signify a significant improvement in subhealth issues within the UMCs.

Furthermore, a high BMI emerges as another prominent driver, particularly in LMCs. Elevated BMI is often associated with low physical activity and unhealthy dietary habits [22]. Building upon the observed trend of low physical activity in the data, this study posits that the relationship between high BMI and dietary health might exhibit even greater significance.

In the analysis of behavioral risk, a relative reduction in tobacco and alcohol use among LMCs resulted in a diminished proportion of behavioral risk. This trend may also indicate an increased awareness of healthcare prevention among individuals in LMCs in recent years. Similarly, a notable decrease in behavioral risk was observed in UMCs, which may be driven by efforts to discourage tobacco consumption. Notably, the previous studies have substantiated the progressive success of tobacco control measures in UMCs [23].

However, the impact of these risk factors is not the same in all LMICs. In sub‐Saharan Africa, for example, countries such as Kenya and Nigeria face significant challenges due to limited healthcare infrastructure and a high prevalence of infectious diseases, which take resources away from non‐communicable diseases such as breast cancer [24]. And these countries also face higher metabolic risks due to rising obesity rates as a result of urbanization and dietary changes [25]. In contrast, South‐East Asian countries such as Vietnam show a different pattern, where genetic predisposition and cultural factors play a more important role in the prevalence of metabolic risk factors [26].

#### 4.1.3 Challenges of LMCs

In this research, we conducted a critical analysis of the factors associated with breast cancer. LMCs may face a more stringent examination compared to other countries. First, the age‐group analysis reveals a peak in DALYs loss, indicating that individuals between the ages of 50 and 55 years are at a higher risk. This result suggests an earlier onset of breast cancer compared to other countries. While considering the highest DALYs cost, it implies that individuals in LMCs may develop breast cancer at younger age but are diagnosed between 50 and 55. The limitation of treatment facilities contributes to the escalation of DALYs, resulting in relatively high cost of DALYs. This unusual phenomenon reflects a severe lack of medical resources, which encompasses, but is not limited to, disease awareness and the universalization of screening practices [27].

The risk factor study has also identified some unusual trends. As mentioned earlier, metabolic risk, including HFPG and BMI, has emerged as a new challenge. Some articles have suggested a positive relationship between HFPG and income [22]. However, according to the classification rules provided by the WB [8], the per capita income of LMCs is higher than that of LCs. Taking a broader perspective, the level of metabolic risk is a more direct indicator of physical health rather than behavioral risk [28]. Therefore, this article concludes that LMCs face greater health pressures.

In cross‐sectional comparative analyses, the mortality annual age‐adjusted percentage changes (AAPCs) for LMCs and LCs were not equal until 2019, and by that year, the mortality AAPC for LMCs surpassed that for LCs. Additionally, the infection rate AAPC for LMCs was significantly higher than that for LCs. In the analysis of AAPCs for the final segments of each group, the AAPC for LMCs was the highest, measuring 1.69 (*T* = 35.4693; *p* < 0.000001). Although some articles have suggested a negative relationship between income and disease burden, this study revealed a partial positive correlation [29].

This article proposes the hypothesis of a nonlinear relationship between LMCs and disease burden in breast cancer. Under the assumption that wealth is negatively correlated with disease stress, LMCs challenge this hypothesis at the national level. Therefore, this article suggests that one possible reason for this phenomenon may be related to external sponsorship, such as assistance from the WHO, which, according to the organization's annual report, is higher for LCs than for LMCs [30]. This may result in a higher level of actual medical resources for LCs than for LMCs. Regardless of the underlying reasons, this article suggests that the presence of nonlinear may be observed in this study, indicating that LMCs may face greater disease pressure.

In addition, differences in construction within LMCs and LCs will also result in domestic risk factors exhibiting different trends. As emphasized by geo‐economists, cities are created to the agglomeration effect of the economy [31]. What this phenomenon symphonized for LMICs is that already limited healthcare resources are concentrated in the country's most developed cities, leading to differences in healthcare resources within the country. This ultimately leads to different risk factor pressures in different parts of the country. This highlights the need for further regionalized data collection and targeted public health interventions to address the specific needs of different regions within LMICs.

### 4.2 Comparison With Previous Literature

In most articles, the classification utilized was based on the socio‐demographic index (SDI), which served as a common framework with this research. However, the aim of this study was to explore categorization on the basis of additional indicators, specifically focusing on indicators used to measure and classify the development or socioeconomic status of countries. Despite this difference in classification approach, the overall trend of the results remained consistent. The findings consistently demonstrated a general pattern or direction, indicating a similarity in the overall outcomes across various classifications. This alignment in the general trend reinforces the robustness and reliability of the results, even while utilizing alternative categorization methods that prioritize quality of life as a key indicator [32, 33, 34].

This article ventures into a less‐explored area by examining the potential association geoculture, as a behavioral factor, and breast cancer. This aspect has received limited attention in previous studies, making it a novel and unique contribution. Additionally, in the context of geographic analysis, this article employs a comparison of ASR with crude rates to extract valuable insights. By utilizing this approach, a clearer understanding of the age distribution of patients with breast cancer in relation to income can be achieved. This method provides a more nuanced perspective on the impact of income disparities on the age profiles of individuals affected by breast cancer. The integration of these approaches enhances the comprehensiveness and depth of the study's findings.

### 4.3 Limitations

In countries with lower levels of medical care, data collection can be less accurate or missing due to various factors. Limited access to diagnostic techniques, healthcare infrastructure, and demographic challenges can impede the collection of precise and comprehensive data [35]. Despite these limitations, infection rates and DALYs loss, albeit imperfect, still provide valuable insights into disease trends and public health burdens. Although the data may not be entirely accurate, they serve as indicators of the general patterns and trends that can inform public health strategies and resource allocation.

It is important to note that the potential relationship of breast cancer, as presented in this article, is just one of the possibilities explored. The study acknowledges that there may be multiple factors at play, which does not claim exclusivity in its findings. It is crucial to consider a range of variables, including genetic predisposition, lifestyle factors, socioeconomic factors, and access to healthcare, among others, to gain a comprehensive understanding of breast cancer etiology [36]. By recognizing the multifactorial nature of breast cancer development, this article contributes to the broader body of knowledge and stimulates further research in this complex field.

### 4.4 Implications

This study provides insights into the potential causes of the burden of breast cancer and highlights the potential application of nonlinear relationship in LMCs. The findings of this study have direct implications for decision‐making regarding the allocation of external resources.

In terms of addressing the burden of breast cancer, the behavioral promotion of smoking cessation emerges as a key factor reflected in risk trends of breast cancer. It is essential for decision‐makers to prioritize and continue implementing interventions in this area. Additionally, decision‐makers should closely monitor metabolic risk factors, as they have the potential to become significant contributors to the burden of breast cancer worldwide.

Future research should consider the influence of geocultural factors and focus on resource distribution within LMCs. Furthermore, it is important to reevaluate the effectiveness of using income class as the sole basis for resource allocation. By considering these aspects, future studies can contribute to more targeted and effective interventions and resource allocation strategies to address the burden of breast cancer in LMCs.

In terms of clinical applications, this study supports early detection programs. Recognizing the significant impact of high HFPG and high BMI on breast cancer risk, people with high metabolic risk factors should be regularly screened as part of routine health assessments in LCs. Early detection programs can be tailored to identify high‐risk populations for early intervention and potentially better outcomes.

The above findings equally imply that LMICs need to further strengthen their healthcare infrastructure, and improvement of facilities could include training of healthcare workers, improving diagnostic tools, and increasing treatment options in underserved areas. The aim is to control the progression of breast cancer at an early stage, with the aim of reducing mortality and improving patients’ QALYs.

## 5 Conclusion

This study provides a comprehensive analysis of the burden of breast cancer in LMICs using the GBD database. Our findings reveal important trends in the prevalence and impact of breast cancer across different demographic and economic groups, with a particular emphasis on the increasing importance of metabolic risk factors such as HFPG and BMI. These data suggest significant changes in risk profiles, with metabolic risk having an increasing impact on the overall burden of disease.

Behavioral risk factors, although historically dominant, show a relative decline in some areas due to increased health awareness and targeted public health interventions. This positive trend highlights the potential of effective prevention programs and educational campaigns to reduce the burden of breast cancer.

However, the challenges remain daunting in LMICs, where limited healthcare infrastructure and resources are a serious impediment to effective disease management and early detection. This study highlights the need for improved healthcare capacity in LMICs regions, including the expansion of early diagnosis and treatment facilities, which is critical to improve patient control of the increasing incidence of metabolic risk‐related breast cancer.

This study also opens up several avenues for future research, particularly exploring the detailed mechanisms by which metabolic risk factors influence breast cancer and how these interactions can be effectively mitigated through clinical and public health strategies. In addition, more in‐depth studies are needed to assess the long‐term effects of current interventions and to develop new strategies that address both behavioral and metabolic risk factors.

## Author Contributions

J.H. and M.C.S.W. conceptualized and supervised this study. J.H., M.G., S.L.W., Q.Y., and F.X. were responsible for data curation, formal analysis, and drafting the manuscript. S.C.C., S.H.C., Y.A.A., C.C.Z., D.E.L.P., and M.C.S.W. made critical revisions to the manuscript and reviewed it.

## Ethics Statement

This study was approved by the Survey and Behavioural Research Ethics Committee, The Chinese University of Hong Kong (No. SBRE‐22‐0826).

## Consent

The authors have nothing to report.

## Conflicts of Interest

Don Eliseo Lucero‐Prisno III, Martin Wong, and Junjie Huang are the members of the editorial board for *Public Health Challenges*. Yusuff Adebayo Adebisi is the member of the youth editorial board for *Public Health Challenges*.

## Supporting information

Supporting Information

## Data Availability

The data used for the analyses are available upon reasonable request from the corresponding author.
