# Supplementary material for: Disease Burden, Risk Factors, and Temporal Trends in Breast Cancer in Low‐ and Middle‐Income Countries: A Global Study
Source: Public Health Chall. 2024 Jul 29;3(3):e223. doi: 10.1002/puh2.223 (PMC12039552; doi:10.1002/puh2.223)
Supplement: Supplementary file 1 — Supporting Information [file PUH2-3-e223-s001.pdf]

Supplementary Table

eTable 1 Risk factor Dependency table

eTable 2 Distribution of the “Orange” in each continent/Percentage

eTable 3 AAPC of breast cancer death rate, 1990-2019

eTable 4 AAPC of breast cancer incidence rate, 1990-2019

eTable 5 AAPC of breast cancer incidence rate, 1990-2019

eTable 1 Risk factor Dependency table

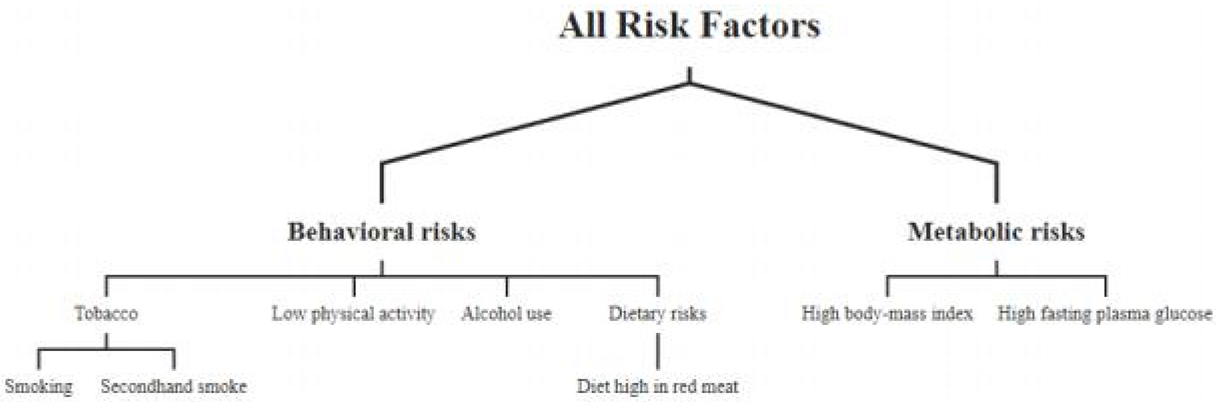

eTable 2 Distribution of the “Orange” in each continent/Percentage

Continent	Compliance countries/Continent countries	Percentage
Europe	0/48	0%
America	10/37	27%
Asia	27/4	56%
Oceania	13/16	81%
Africa	47/53	87%

eTable 3 Religious population of distribution in "Orange" counties

First in number of believers (Number of counties)	Second in number of believers (Number of counties)
Islam (38)	Islam (40)
Christianity (38)	Christianity (31)
Buddhist (3)	Buddhist (5)
Hinduism (2)	Hinduism (5)

eTable 4 AAPC of breast cancer death rate, 1990-2019

	1					2					3					4					5					6							
	Segment Start	Segment End	APC	Test Statistic	P-Value	Segment Start	Segment End	APC	Test Statistic	P-Value	Segment Start	Segment End	APC	Test Statistic	P-Value	Segment Start	Segment End	APC	Test Statistic	P-Value	Segment Start	Segment End	APC	Test Statistic	P-Value	Segment Start	Segment End	APC	Test Statistic	P-Value	AAPC	Test Statistic	P-Value
High Income	1990 1995		-0.69	-7.167	0.000001	1995 2007		-1.568	-54.2389	< 0.000001	2007 2014		-1.219	-16.796	< 0.000001	2014 2019		-0.676	-7.0193	0.000001											-1.179	-37.3057	< 0.000001
Upper Middle Income	1990 1994		1.646	1.2942	0.209005	1994 1997		-0.863	27.123	< 0.000001	1997 2003		0.609	42.0058	< 0.000001	2003 2013		-0.815	20.0311	< 0.000001	2013 2019		-0.343	-1.1768	0.260359						-0.092	-0.8363	0.402993
Lower Middle Income	1990 1997		1.269	-1.9757	0.069813	1997 2002		-0.175	2.2618	0.041493	2002 2005		-0.927	3.2517	0.006307	2005 2012		0.18	2.3319	0.036436	2012 2015		1.544	6.1066	0.000015	2015 2019		0.348	-1.0247	0.320727	0.429	5.3332	< 0.000001
Low 1.1 Income 64	1990 0.000	1994 2009		2019	0.145	3.2121		0.005437	- 1994 052	< 2009 001			0.4 10.651	2.4026	0.028771		-														0.654	32.502	< 0.000001

eTable 5 AAPC of breast cancer incidence rate, 1990-2019

	1					2					3					4					5					6							
	Segment Start	Segment End	APC	Test Statistic	P-Value	Segment Start	Segment End	APC	Test Statistic	P-Value	Segment Start	Segment End	APC	Test Statistic	P-Value	Segment Start	Segment End	APC	Test Statistic	P-Value	Segment Start	Segment End	APC	Test Statistic	P-Value	Segment Start	Segment End	APC	Test Statistic	P-Value	AAPC	Test Statistic	P-Value
High Income	1990 1.7	1994		10.1653	< 0.000001	1994	2003	0.255	4.4464	0.000659	2003 2006		-0.809	-1.5498	0.145189	2006	2009	0.361	0.6863	0.504573	2009 2014		-0.805	-4.8758	0.000303	2014 2019		-0.393	-3.3577	0.005144	0.057	0.643	0.520206
Upper Middle Income	1990 2009		1.925	43.8603	< 0.000001	2009	2019	1.277	11.1073	< 0.000001																					1.701	34.696	< 0.000001
Lower Middle Income	1990 1997		1.743	18.4354	< 0.000001	1997	2008	0.401	7.3287	< 0.000001	2008 2019		1.691	35.4693	< 0.000001																1.212	33.9149	< 0.000001
Low Income	1990 1997		0.417	9.159	< 0.000001	1997	2009	0.848	36.8206	0.000001	2009 1.6	2019		< 59.9401	0.000001								<								1.002	58.1783	< 0.000001
